# The wheat R2R3-MYB transcription factor TaRIM1 participates in resistance response against the pathogen *Rhizoctonia cerealis* infection through regulating defense genes

**DOI:** 10.1038/srep28777

**Published:** 2016-07-01

**Authors:** Tianlei Shan, Wei Rong, Huijun Xu, Lipu Du, Xin Liu, Zengyan Zhang

**Affiliations:** 1 The National Key Facility for Crop Gene Resources and Genetic Improvement, Institute of Crop Science, Chinese Academy of Agricultural Sciences, Beijing 100081, China

## Abstract

The necrotrophic fungus *Rhizoctonia cerealis* is a major pathogen of sharp eyespot that is a devastating disease of wheat (*Triticum aestivum*). Little is known about roles of MYB genes in wheat defense response to *R. cerealis*. In this study, *TaRIM1*, a *R. cerealis*-induced wheat MYB gene, was identified by transcriptome analysis, then cloned from resistant wheat CI12633, and its function and preliminary mechanism were studied. Sequence analysis showed that *TaRIM1* encodes a R2R3-MYB transcription factor with transcription-activation activity. The molecular-biological assays revealed that the TaRIM1 protein localizes to nuclear and can bind to five MYB-binding site *cis*-elements. Functional dissection results showed that following *R. cerealis* inoculation, *TaRIM1* silencing impaired the resistance of wheat CI12633, whereas *TaRIM1* overexpression significantly increased resistance of transgenic wheat compared with susceptible recipient. TaRIM1 positively regulated the expression of five defense genes (*Defensin*, *PR10*, *PR17c*, *nsLTP1*, and *chitinase1*) possibly through binding to MYB-binding sites in their promoters. These results suggest that the R2R3-MYB transcription factor TaRIM1 positively regulates resistance response to *R. cerealis* infection through modulating the expression of a range of defense genes, and that *TaRIM1* is a candidate gene to improve sharp eyespot resistance in wheat.

Common wheat (*Triticum aestivum* L.) is one of most important staple crops in the world and plays a fundamental role in food security. Wheat production often is negatively affected by various fungal diseases. The sharp eyespot disease, mainly caused by the soil-borne fungus *Rhizoctonia cerealis* van der Hoeven (teleomorph: *Ceratobasidium cereale* D. Murray & L.L. Burpee), is a devastative disease of wheat production in the world^1^,^2^,^3^. The sharp eyespot symptoms in wheat include dark-bordered lesions on stem bases and based sheaths of adult-plants^3^. The sharp eyespot disease can destroy the transport tissues in stems of plants, and cause pre- and post-emergence damping off and premature spike senescence or ripening (white heads), leading to yield losses of ~10–30%. The environmentally safe and efficient way to protect wheat from sharp eyespot is to breed resistant wheat varieties. However, the resistances in partially-resistant wheat lines are controlled by multiple quantitative loci^1^,^4^. Currently, breeding sharp eyespot-resistant wheat cultivars using traditional methods is difficult since none of sharp eyespot-immune wheat cultivars/lines is available. Thus, to improve wheat resistance to *R. cerealis*, it is necessary to identify important genes in the defense response and clarify their defense roles.

In order to defend attack of pathogens, plants have evolved comprehensive and complicated defense system. The transcription factor families play an important role in the plants responses against different stresses. The myeloblastosis (MYB) transcription factors are characterized by a DNA-binding MYB domain. The MYB domain is composed of approximately 52 amino acid residues that adopt a helix-turn-helix conformation that intercalates into the major groove of DNA^5^,^6^. Since the first plant MYB gene *COLORED1* (*C1*) being involved in anthocyanin biosynthesis was identified in maize^7^, a large number of MYB proteins have been identified in different plant species. Based on the number of adjacent MYB repeats, MYB proteins can be divided into 4 classes: 1R-MYB, 2R-MYB (R2R3-MYB), 3R-MYB and 4R-MYB^6^. Numerous MYB proteins have been implicated in diverse biological processes, including cell cycle regulation, cell wall biosynthesis, development and reproduction, and defense responses to abiotic and biotic stresses^6^,^8^,^9^,^10^,^11^,^12^,^13^,^14^. To date, most of the identified MYB genes belong to R2R3-MYB subfamily, and a number of R2R3-MYB transcriptional factors have been evidenced to play different important roles in various plant species^6^,^15^,^16^,^17^,^18^,^19^,^20^,^21^. For example, in Arabidopsis, *BOS1* (*BOTRYTIS-SUSCEPTIBLE1*), an R2R3-MYB gene (AtMYB108), is required for restricting the spread of 2 necrotrophic pathogens *Botrytis cinerea* and *Alternaria brassicicola*, and involved in the tolerance to osmotic and oxidative stresses^22^. Overexpression of an *Arabidopsis* R2R3-MYB AtMYB96 could enhance tolerance to drought stress^23^ and increase resistance to bacteria pathogens^24^. In barley, the MYB transcription factor HvMYB6 functions as positive regulator of basal and MLA-mediated immunity responses to *Blumeria graminis*^25^. Ectopic expression of the wheat MYB gene *TaMYB33* that was induced by NaCl and PEG stresses increased salt and drought tolerance in Arabidopsis plants^17^. The ectopic expression of TaMYB73 improved salt tolerance of transgenic Arabidopsis plants^15^. Overexpression of the wheat pathogen-induced MYB gene *TaPIMP1* in transgenic wheat could significantly enhance resistance to the fungal pathogen *Bipolaris sorokiniana* and drought stresses^26^. Ectopic expression of a *Thinopyrum intermedium* MYB gene *TiMYB2R-1* could significantly increase resistance of transgenic wheat lines to take-all caused by *Gaeumannomyces graminis*^27^. Silencing of a wheat R2R3-MYB gene *TaMYB4* in wheat impaired the resistance to *Puccinia striiformis* f. sp. *tritici*^5^. However, none of MYB genes being involved in defense response to *R. cerealis* infection has been reported yet.

In this study, we identified and functional characterized a *R. cerealis*-induced MYB gene in wheat, named *TaRIM1*. Toward *R. cerealis* infection, the gene expression goes higher level. The sequence analysis and bio-molecular assays proved that TaRIM1 protein is a R2R3-type MYB transcription factor. It is localized in the nucleus and can bind to MYB binding site *cis*-elements. Through generation of *TaRIM1*- silencing and overexpression wheat plants and assessment of their defense responses following *R. cerealis* inoculation, the functional dissection results indicated that TaRIM1 positively modulated wheat defense response to *R. cerealis*. Further investigation suggested that TaRIM1 might activate the expression of a range of defense-related genes, resulting in enhanced resistance to *R. cerealis* infection.

## Results

### Identification and cloned sequence of *TaRIM1* induced by *R. cerealis* infection

**Figure 1:**
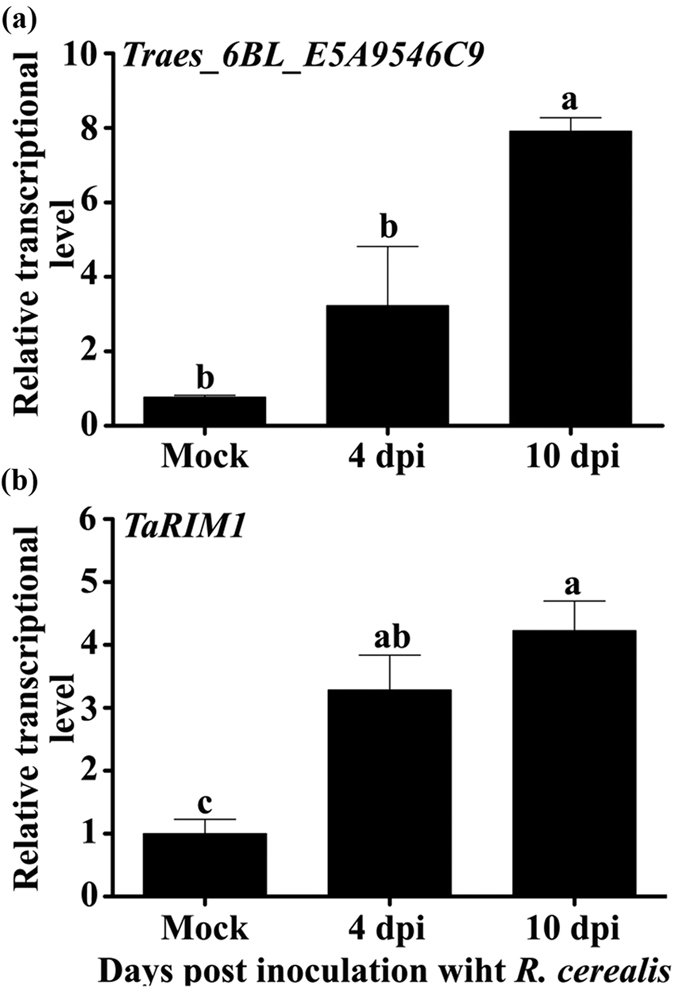
Transcriptonal analyses of *TaRIM1* in *Rhizoctonia cerealis*-inoculated wheat. The RNAs are isolated from pooled stems of 3 resistant lines in recombinant inbred lines of cross of *R. cerealis*-resistant wheat Shanhongmai and susceptible wheat cultivar Wenmai 6 at 4, or 10 days post inoculation (dpi) with *R. cerealis* strain WK207 or mocked ones. (**a**) RNA-Seq data of the sequence Traes_6BL_E5A9546C9 being corresponding to *TaRIM1*. (**b**) qRT-PCR verification of the transcription of *TaRIM1*. The transcriptional level in the mocked one is set to 1. The transcriptional levels with different letters are significantly different from each other based on statistically significant analysis on the results of three replications (*t*-test, **P < 0.01).

To identify wheat genes being involved in defense response to *R. cerealis*, we performed transcriptomic analysis through Deep RNA-seq on 3 resistance lines of the recombinant inbred lines (RILs, being derived from the cross of sharp eyespot-resistant wheat line Shanhongmai and sharp eyespot-susceptible wheat cultivar Wenmai 6) at 4 and 10 d post inoculation (dpi) with *R. cerealis* high-virulence strain WK207 (Unpublished). Among the up-regulated sequences, the expression of the sequence with no. Traes_6BL_E5A9546C9, being homologous to the wheat MYB gene *TaMYB33* sequence, was up-regulated in the resistant wheat lines after *R. cerealis* inoculation. It showed a 4.18-fold at 4 dpi or a 10.23-fold at 10 dpi transcriptional increase than the mocked (Fig. 1a). Quantitative RT-PCR (qRT-PCR) analysis showed that the transcriptional levels of this gene were induced after *R. cerealis* inoculation (Fig. 1b), and the expression tendency by experimental qRT-PCR was in agreement with the RNA-Seq data. This gene was designated as *TaRIM1* and was suggested to be involved in wheat defense response to *R. cerealis* infection.

**Figure 2:**
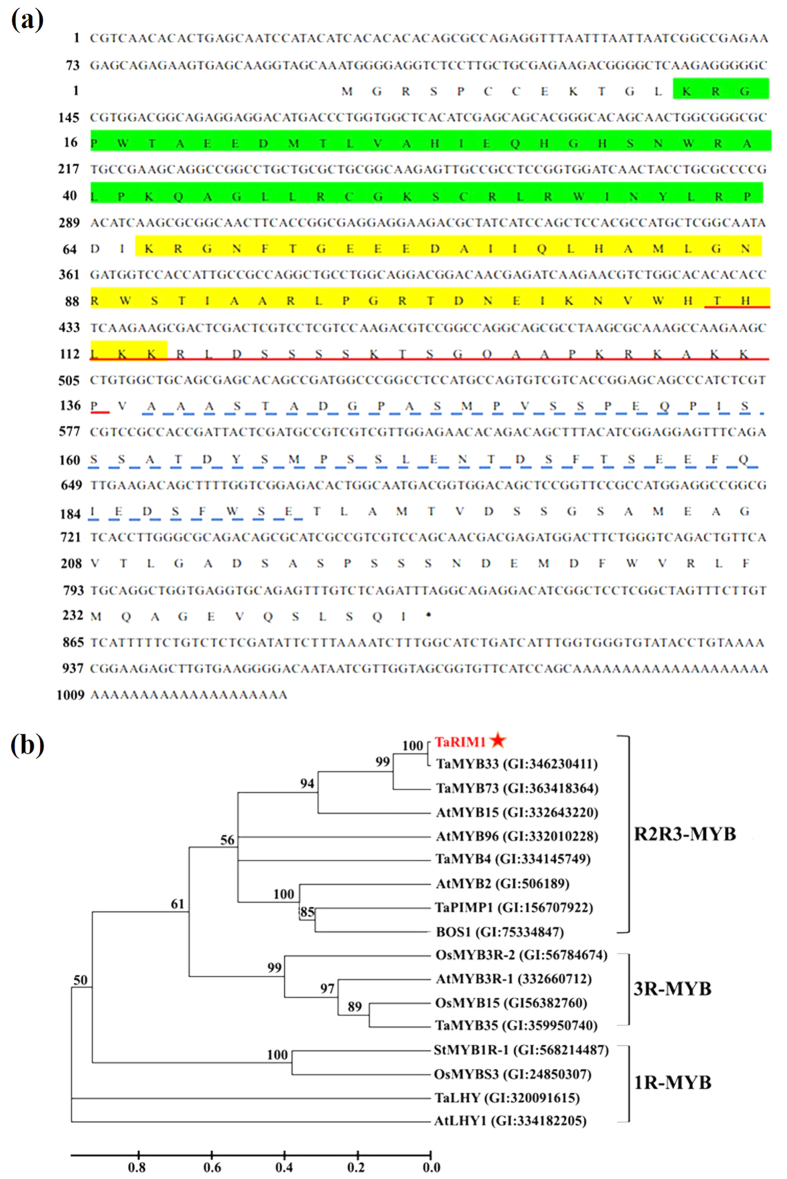
The sequence and phylogenetic tree analyses of TaRIM1. (**a**) The nucleotide sequence and deduced amino acid sequence of TaRIM1. Green part represents R2 domain, yellow space indicates R3 domain, the nucleus localization signal is marked by red lines, and blue dotted line represents the acidic region. (**b**) Phylogenetic analysis of the TaRIM1 protein. The phylogenetic tree including TaRIM1 protein and 16 known-function MYB proteins is constructed using neighbor-joining phylogeny of MEGA 5.0, and is showed in bootstrapped manner.

The full-length cDNA sequence (with 1028 bp, Fig. 2a, NCBI accession no. KU864997) of *TaRIM1* was obtained from *R. cerealis*-infected stem cDNA of the resistance wheat line CI12633 by RACE and nest RT-PCR. It includes the complete ORF with 732-bp, 5′-untranslated region (UTR) with 98-bp, and 3′-UTR with 198-bp. The genomic DNA sequence of *TaRIM1* was amplified from CI12633 genomic DNA. The comparison of the genomic and cDNA sequences indicated that no intron existed in genomic transcription unit of *TaRIM1*, at least in the amplification region. The deduced protein TaRIM1 contains 243 amino acids with a predicted molecular weight of 26.61 KD and predicted PI of 6.09. As shown in Fig. 2a, the TaRIM1 protein sequence possesses two conserved MYB DNA-binding domains [one (R2) located at amino acids 13-63 and another (R3) at amino acids 66-114], a putative nuclear localization signal (NLS, located at amino acids110-136), and an acidic region (amino acids 138-191) possibly acting as a transcription activation domain^28^. The reconstructed phylogenetic tree analysis showed that this protein TaRIM1 was clustered into the R2R3-MYB subfamily (Fig. 2b). Thus, TaRIM1 most likely is a R2R3-MYB transcription factor with transcription-activation activity.

### TaRIM1 localizes to the nucleus and binds to MYB-binding site *cis*-elements

**Figure 3:**
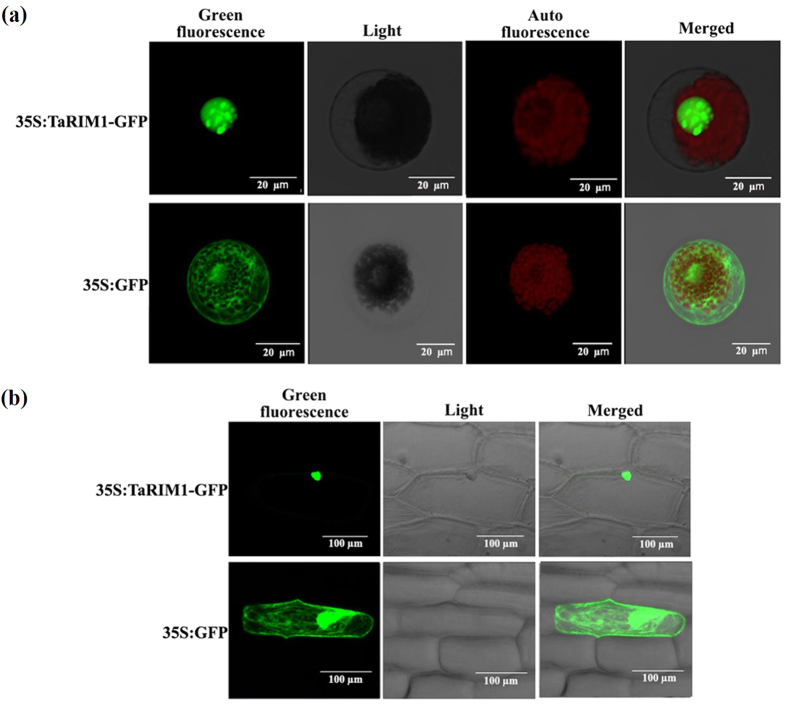
Subcellular localization of the TaRIM1 protein in either the wheat mesophyll protoplasts or onion epidermal cells. The 35S:TaRIM1-GFP and 35S:GFP constructs were separately introduced into and transiently expressed in both the wheat mesophyll protoplasts (**a**) and onion epidermal cells (**b**). The transient expressing green-fluorescence proteins (GFPs) were observed and photographed under 488 nm in Confocal Laser Scanning Microscopy (Zeiss LSM 700, Germany). Auto-fluorescence of the wheat chloroplast was observed and photographed under 639 nm in Confocal Laser Scanning Microscopy (Zeiss LSM 700, Germany).

As the TaRIM1 protein sequence contains the NLS sequence (Fig. 2a), the p35S:TaRIM1-GFP (green fluorescent protein) fusion expressing-vector was prepared for investigating the subcellular localization of TaRIM1. The p35S:TaRIM1-GFP and control p35S:GFP construct DNAs were separately introduced into and transiently expressed in both wheat mesophyll protoplasts and onion epidermal cells. Confocal imaging of the transient expression showed that TaRIM1-GFP localized in the nucleus in both the wheat mesophyll protoplasts and onion epidermal cells (Fig. 3a,b), whereas the fluorescence of the control GFP was distributed throughout the cell (Fig. 3a,b). These results indicated that the expressing TaRIM1 protein localizes in the nuclear.

**Figure 4:**
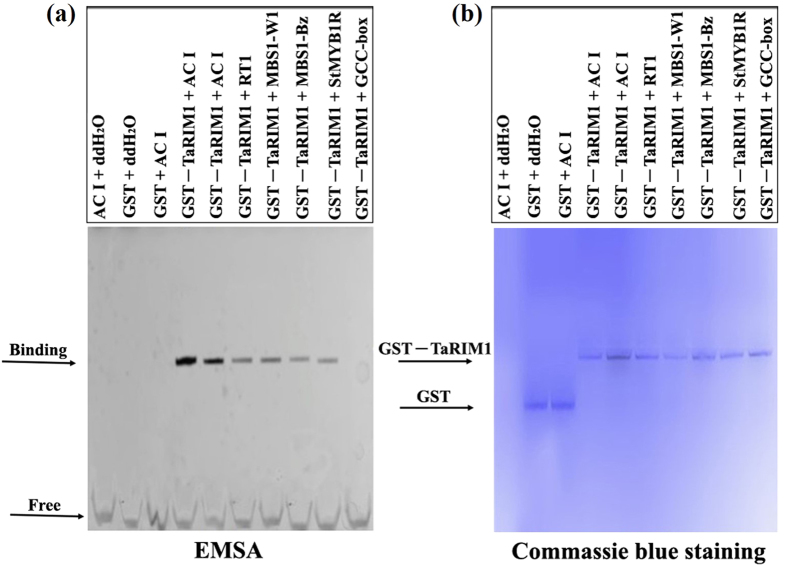
Electrophoretic mobility shift assay on binding activity of TaRIM1 to MBS *cis*-element. MBS indicates MYB-binding site. (**a**) The retarded band of binding of GST-TaRIM1 fusion protein to MBS probe and free probe are indicated by arrows. (**b**) Commassie blue staining shows the position of GST protein and GST-TaRIM1 fusion protein by arrows.

The amino acid sequence of TaRIM1 contains R2 and R3 DNA-binding domains. To investigate if TaRIM1 binds to MYB-binding site (MBS) *cis*-elements, the glutathione *S*-transferase (GST)-TaRIM1 recombinant protein was prepared, expressed, and purified. Electrophoretic mobility shift assay (EMSA) was used to examine the DNA binding ability of TaRIM1 with MBS. Here, the tested 5 MBS *cis*-elements include ACI, MBS1-Bz, MBS1-w, RT1, and St1R that were bound by known functional R2R3-MYB, StMYB1R-1 and OsMYB3R-2 transcription factors^26^ (Fig. 4a, Table S1). The EMSA results showed that the GST-TaRIM1 protein could bind to all the tested 5 MBS probes, especially showing the strongest binding to ACI, but not bind to the GCC-box *cis*-element that is specifically bound by ERF transcription factors, whereas GST failed to bind with the MBS *cis*-element ACI and the GCC-box *cis*-element (Fig. 4b). These data proved that the protein TaRIM1 can bind to these five MBS elements.

### Silencing of *TaRIM1* impairs wheat resistance to *R. cerealis*

**Figure 5:**
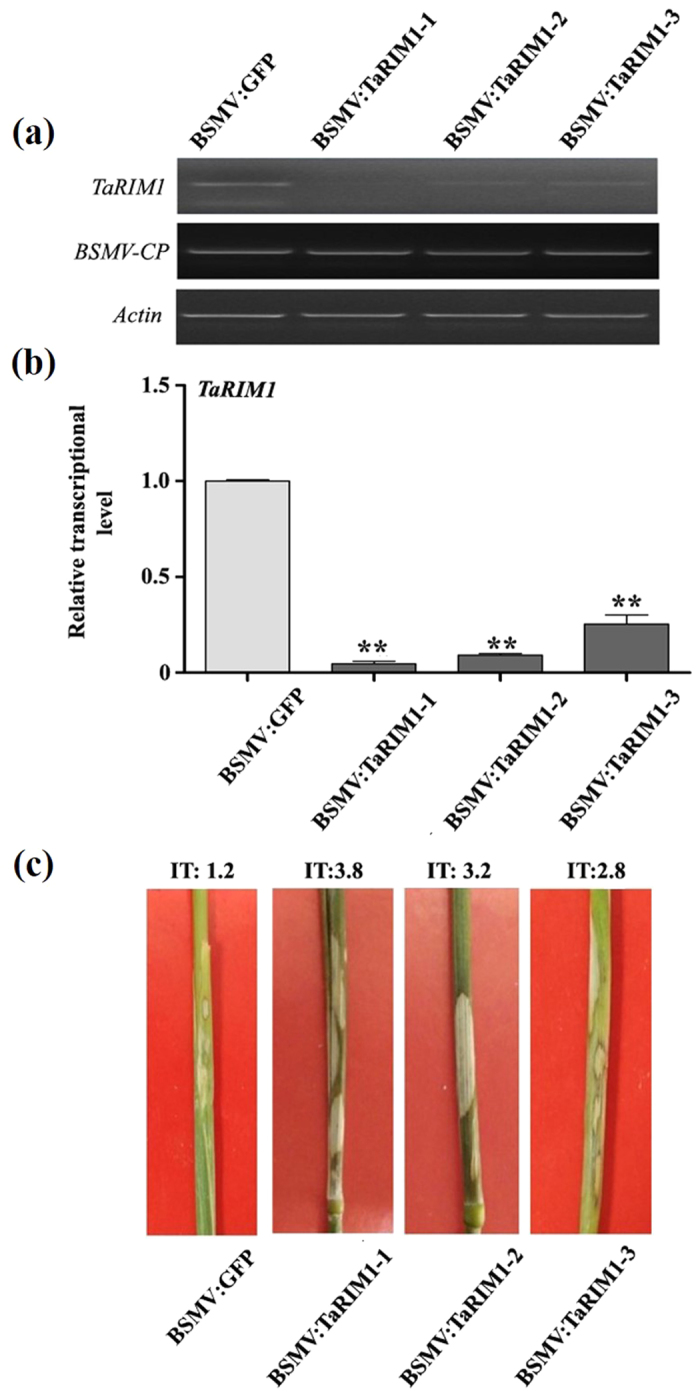
VIGS-based functional analysis of TaRIM1 in response of wheat to *Rhizoctonia cerealis*. (**a**) RT-PCR analysis of the transcription levels of *TaRIM1* and the barley stripe mosaic virus (BSMV) *CP* gene in wheat plants infected by BSMV:GFP or BSMV:TaRIM1 for 15 d. (**b**) qRT-PCR analysis of relative transcript level of *TaRIM1* in stems of wheat CI12633 plants infected by BSMV:TaRIM1 or BSMV:GFP at 45 dpi with *R. cerealis* strain WK207. The relative transcript level of *TaRIM1* in BSMV:TaRIM1-infected wheat plants is relative to that in BSMV:GFP-infected plants. Statistically significant differences between BSMV:GFP**-infected and BSMV:TaRIM1-infected (*TaRIM1*-silencing) CI12633 plants were determined based on three replications using Student’s *t*-test (**P < 0.01). (**C**) Typical sharp eyespot phenotypes of *TaRIM1*-silencing CI12633 plants and BSMV:GFP-infected CI12633 plants after inoculation with *R. cerealis* strain WK207 for 45 days. IT indicates infection type of wheat plants to *R. cerealis* infection.

Virus-induced gene silencing (VIGS) is an efficient reverse-genetic tool for rapidly analyzing functions of genes in plants. Barley stripe mosaic virus (BSMV)-based VIGS is extensively used for investigating functions of interest genes in barley and wheat^29^,^30^,^31^. To explore whether *TaRIM1* plays an important role in wheat resistance response against *R. cerealis*, we used BSMV-based VIGS method to down-regulate transcriptional levels of *TaRIM1* in the resistant wheat line CI12633. At 15 dpi with the virus, the transcript of BSMV coat protein (*cp*) gene was readily detected (Fig. 5a), suggesting that BSMV successfully infected these wheat plants. Importantly, the transcript levels of *TaRIM1* were significantly reduced in CI12633 plants infected by BSMV:TaRIM1 compared to BSMV:GFP infected CI12633 plants (control plants) (Fig. 5a,b), suggesting that *TaRIM1* transcript was successfully down-regulated in BSMV:TaRIM1 infected plants, hereafter *TaRIM1*-silenced plants represented BSMV:TaRIM1 infected ones. The *TaRIM1*-silenced and BSMV:GFP infected CI12633 plants were further inoculated with *R. cerealis*. Subsequently, the infection types (ITs) by the fungus were evaluated. At 45 dpi with *R. cerealis*, *TaRIM1*-silenced CI12633 plants showed more susceptible to the sharp eyespot disease caused by *R. cerealis* (ITs: ~2.8–3.8; Fig. 5c), whereas BSMV:GFP infected CI12633 plants showed more resistance of sharp eyespot (IT: 1.2, Fig. 5c). These results suggested the down-regulation of *TaRIM1* compromised the resistance to *R. cerealis* in CI12633, and that *TaRIM1* is required for host resistance response to *R. cerealis*.

### Overexpression of *TaRIM1* enhances wheat resistance to *R. cerealis*

**Figure 6:**
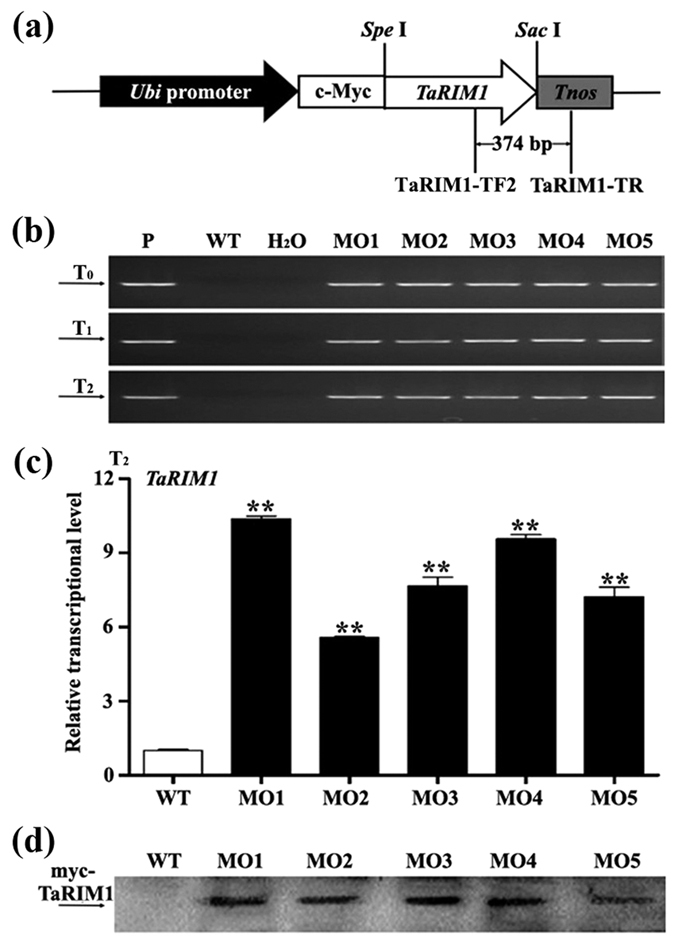
The transformation vector and molecular characterization of *TaRIM1*-overexpressing transgenic wheat plants. (**a**) The *TaRIM1*-overexpression transformation vector pUbi:myc-TaRIM1. The arrow indicates the fragment amplified in the PCR detection of the transgene. (**b**) PCR patterns of T_0_-T_2_ plants. P: indicates pUbi:myc-TaRIM1 plasmid as the positive control; MO1-MO5 indicate 5 *TaRIM1* transgenic wheat lines. WT indicates non-transformation wheat (recipient) Yangmai 16. (**c**) qRT-PCR analysis of *TaRIM1* transcriptional levels in these 5 transgenic wheat lines. The relative transcript level of *TaRIM1* in these transgenic lines is relative to that in the WT plants. Three biological replicates each line were averaged and statistically treated (*t*-test; **P < 0.01). Bars indicate standard error of the mean. (**d**) Western blot pattern of these 5 *TaRIM1*-overexpressing transgenic lines and WT Yangmai 16 using an anti-myc antibody.

To further investigate the defense role of *TaRIM1* in wheat, we constructed the *TaRIM1* overexpression vector pUbi:myc-TaRIM1 (Fig. 6a), in which the expression of the fused protein gene *myc-TaRIM1* of a *c-myc* epitope tag and *TaRIM1* was driven by a maize ubiquitin (*Ubi*) promoter and terminated by the terminator of the *Agrobacterium tumefaciens* nopaline synthase gene (*Tnos*) in a modified monocot transformation vector pAHC25 31,32. The pUbi:myc-TaRIM1 vector DNA was bombarded by gene gun into immature embryos of the spring wheat cultivar Yangmai 16 for generating transgenic wheat plants. The presence of *TaRIM1* transgene cassette was detected by the desired PCR product (374 bp) using the primer pairs specific to *TaRIM1-Tnos* transgene (Fig. 6b). Based on results of PCR detection in 3 successive generations of T_0_-T_2_, five stably transgenic lines (MO1-MO5) containing *Ubi:myc*-*TaRIM1* transgene were selected. qRT-PCR assays showed that the transcriptional levels of *TaRIM1* in these five *TaRIM1*-overexpressing transgenic lines were significantly elevated compared with non-transformed (wild type, WT) recipient wheat Yangmai 16 (Fig. 6c). Western blotting analysis indicated that the introduced *myc-TaRIM1* gene was translated into the myc-TaRIM1 protein in these 5 overexpressing transgenic lines (MO1-MO5), but not in WT Yangmai 16 (Fig. 6d). Following inoculation with *R. cerealis* for ~47 d, all the 5 *TaRIM1*-overexpressing wheat lines in successive two (T_1_-T_2_) generations showed significantly enhanced-resistance to sharp eyespot compared with susceptible WT wheat Yangmai 16 (Table 1). These results indicated that TaRIM1 positively contributed to wheat defense response to *R. cerealis* infection.

### TaRIM1 positively regulates the expression of defense genes in wheat

**Figure 7:**
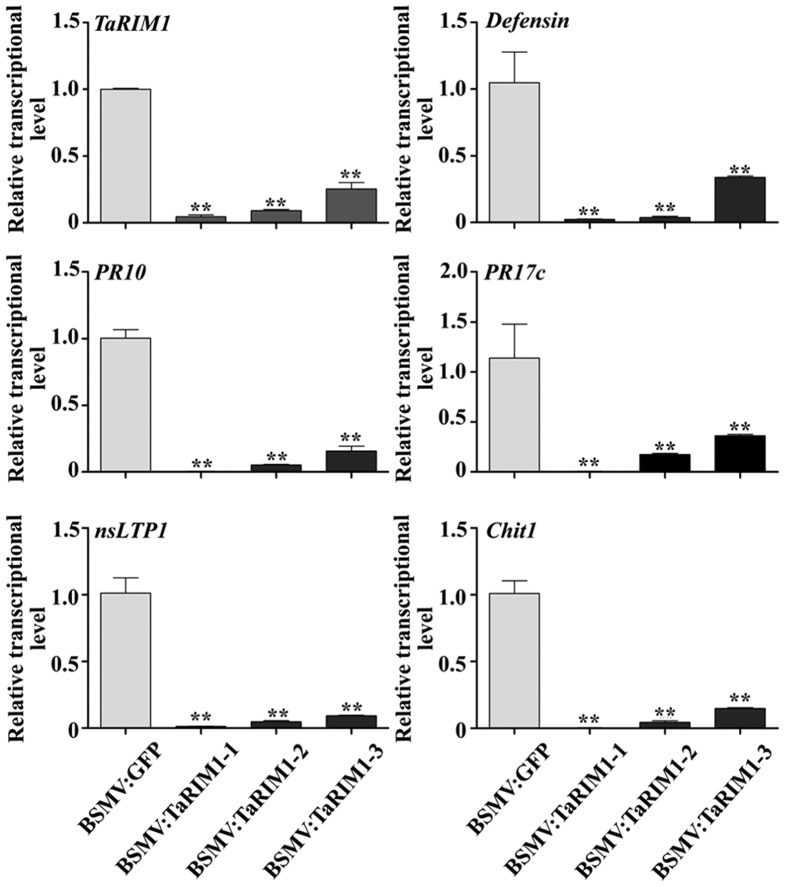
qRT-PCR analysis of the relative transcript level of *TaRIM1* and five defense genes in *TaRIM1*-silencing and BSMV:GFP-infected CI12633 plants inoculated with *R. cerealis* for 45 d. The relative transcript level of the tested genes in *TaRIM1*-silencing wheat CI12633 plants is relative to that in BSMV:GFP-infected CI12633 plants. The examined genes include *Defensin* (NCBI accession no. CA630387), *PR10* (NCBI accession no. CA613496), *PR17*c (NCBI accession no. TA65181), *nsLTP1* (NCBI accession no. TC411506), and *chitinase1* (*Chit1*, NCBI accession no. CA665185). Statistically significant differences between *TaRIM1*-silencing wheat plants and BSMV:GFP-infected plants were determined based on three replications using Student’s *t*-test (**P < 0.01).

**Figure 8:**
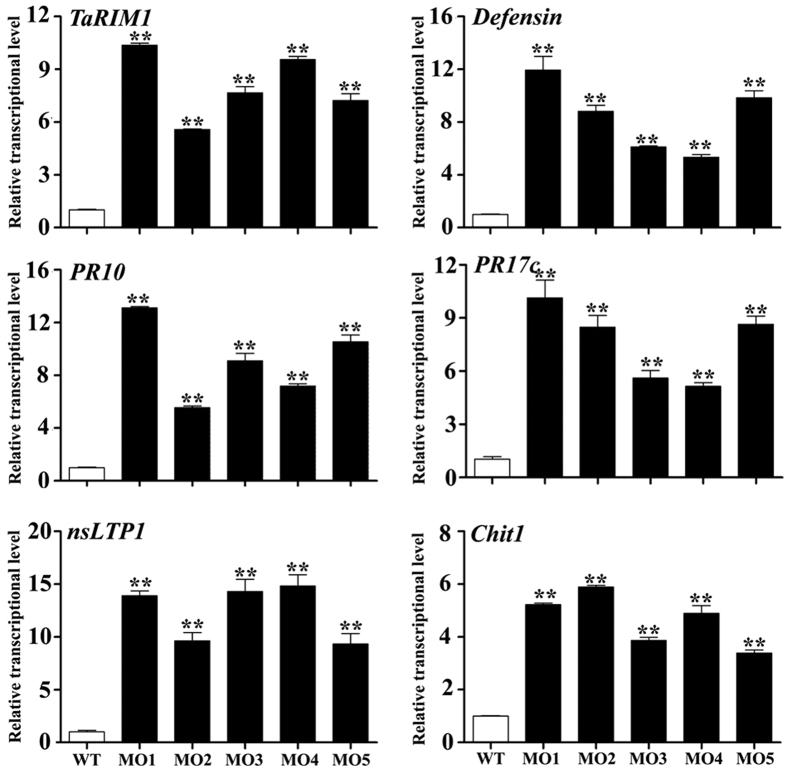
qRT-PCR analysis of the expression of *TaRIM1* and five defense genes in *TaRIM1*-overexpressing transgenic and WT lines inoculated with *R. cerealis* for 47 d. The relative transcript level of the tested genes in transgenic lines is relative to that in the WT plants. The examined genes include *Defensin* (NCBI accession no. CA630387), *PR10* (NCBI accession no. CA613496), *PR17*c (NCBI accession no. TA65181), *nsLTP1* (NCBI accession no. TC411506), and *chitinase1* (*Chit1*, NCBI accession no. CA665185). Statistically significant differences between transgenic lines with WT were determined based on three replications using Student’s *t*-test (**P < 0.01).

To explore the putative mechanism of *TaRIM1* in the resistance response to *R. cerealis* infection, we analyzed the transcript levels of 5 wheat defense genes by qRT-PCR in *TaRIM1*- silencing and overexpression wheat plants as well their controls after the pathogen inoculation. The examined genes include *Defensin* (NCBI accession no. CA630387), *PR10* (NCBI accession no. CA613496), *PR17*c (NCBI accession no. TA65181), *nsLTP1* (NCBI accession no. TC411506), and *chitinase1* (*Chit1*, NCBI accession no. CA665185). As shown in Fig. 7, the transcription levels of all the 5 defense genes were significantly decreased in susceptible *TaRIM1*-silenced wheat plants than that in BSMV:GFP infected control plants. These results suggested that the expression level of *TaRIM1* was correlated with the transcriptional levels of these defense genes. As expected, the transcription levels of these 5 defense genes were significantly elevated in resistant *TaRIM1*-overexpressing transgenic wheat lines compared to those in susceptible WT wheat Yangmai 16 plants (Fig. 8). These results indicated that TaRIM1 positively regulated, most likely activated, the expression of these defense genes in wheat.

MYB proteins can regulate the expression of defense- and stress-related genes following by interaction with MBS *cis*-elements. Our above EMSA results showed that the TaRIM1 protein could bind to 5 MBS motif sequences (Fig. 4). Furthermore, to address how TaRIM1 activates the afore-tested 5 defense genes, we obtained the promoters’ sequences of these 5 defense genes from International Wheat Genome Sequencing Consortium (http://www.wheatgenome.org/), and then searched MBS *cis*-elements in −2000 to −1 bp promoter sequences upstream of ATG of all of these genes using PLACE (https://sogo.dna.affrc.go.jp/cgi-bin/sogo.cgi?lang=en&pj=640&action=page&page=newplace) and the tested 5 MBS motif sequences. As shown in Table 2, the promoters of *Defensin* and *PR10* contain 4 MBS motif sequences, including ACI, MBS1, RT1, and St1R, respectively. The promoter of *Chit1* contains 3 MBS motif sequences, including ACI, MBS1, and RT1. The promoter of *PR17*c and *nsLTP1* contain 2 MBS *cis*-elements, including MBS1 and St1R. These data implied that TaRIM1 may interact with these MBS *cis*-elements in the promoters of these defense genes.

## Discussion

The sharp eyespot disease, caused primarily by the necrotrophic fungal pathogen *R. cerealis*, seriously limits the wheat production worldwide. The wheat defense response to *R. cerealis* is complicated and involves expression changes of a series of defense-related genes^31^,^33^. Identification of important genes in wheat defense response to *R. cerealis* is critical for developing wheat varieties with resistance to sharp eyespot. In plants, MYB transcription factors play important roles in development and defense responses against abiotic and biotic stresses. Many MYB genes are induced after various stress stimuli. For example, the Arabidopsis R2R3-MYB gene *BOS1* showed significant induction after *B. cinerea*, *bos1* mutant displayed more sensitivity to 2 necrotrophic pathogens, osmotic and oxidative stresses than WT plants^22^. In wheat, the R2R3-MYB gene *TaPIMP1* was induced after *B. sorokiniana* infection and drought stress, and positively regulated resistance responses to *B. sorokiniana* and drought stresses^26^. *TaLHY*, a wheat 1R-MYB gene, was induced by the infection of stripe rust pathogen strain CYR32. VIGS-based functional analysis suggest that *TaLHY* may positively participate in wheat defense response to the biotrophic fungal pathogen CYR32 13. However, no MYB gene being involved in resistance to the necrotrophic pathogen *R. cerealis* has been identified yet.

In this study, through RNA-Seq-based transcriptomic analyses, *TaRIM1*, a wheat MYB gene induced by *R. cerealis* in wheat, was identified, and cloned from *R. cerealis*-resistant wheat CI12633. In fact, some stress-induced genes play important roles in defense responses to the stresses, whereas other stress-induced genes do not. To explore the functional role of *TaRIM1* in wheat defense response to *R. cerealis*, we generated the *TaRIM1*- silencing and overexpression wheat plants. Through molecular characterization and assessment of resistance responses of *TaRIM1*- silencing and overexpression as well their control wheat plants after inoculation with the fungal pathogen, the functional dissection results displayed that silencing of *TaRIM1* did obviously impair resistance to *R. cerealis* in CI12633, *TaRIM1*-overexpression wheat lines exhibited significantly enhanced-resistance compared with susceptible WT recipient Yangmai16. These data suggest that *TaRIM1* is required for defense response against *R. cerealis* in wheat, at least for the resistant wheat line CI12633, and positively contributes to wheat defense response to *R. cerealis* infection. Moreover, the transgenic wheat produced in this study will provide potential wheat germplasm for enhancing resistance to sharp eyespot disease. To our knowledge, TaRIM1 is the first reported member of MYB family positively participating in resistance response to *R. cerealis*.

In this report, Blast and phylogenetic analyses showed that the deduced protein TaRIM1 is a member of the R2R3-MYB subfamily. The sequence of the TaRIM1 protein possesses R2 and R3 MYB DNA-binding domains, a NLS and a putative transcription-activation domain, implying that the TaRIM1 protein is an activator-type R2R3-MYB transcription factor. Our biochemical and molecular-biological experiment results reveal that TaRIM1 is localized in the nuclear and can bind to all the test 5 MBS *cis*-elements. These biochemical properties are in agreement with the sequence characteristics of TaRIM1, and are necessary for the active function of MYB transcription factors^5^.

In plants, activator-type transcription factors have been implicated in defense responses through activating the expression of defense-related genes^10^,^26^,^27^,^28^,^33^,^34^,^35^. Defense genes positively contribute to resistance to pathogens in plants^24^,^26^. For example, transgenic wheat plants overexpressing a barley *chitinase* gene or a radish defensin gene *RsAFP2* or a wheat *LTP* gene showed enhanced-resistance to fungal pathogens^36^,^37^,^38^. To explore the putative mechanism of TaRIM1 in wheat resistance response, we analyzed the transcriptional levels of 5 wheat defense genes, including *Defensin, PR10, PR17*c, *nsLTP1*, and *Chit1*, in *TaRIM1*-silencing and *TaRIM1*-overexpression wheat plants and their control plants. The results showed that the expression levels of these 5 defense genes were lower in *TaRIM1*-silenced wheat than in the control plants, whereas the transcriptional levels of the 5 genes in *TaRIM1*-overexpression wheat plants were elevated compared with non-transformed recipient wheat, suggesting that TaRIM1 positively regulates the transcriptional levels of the above-tested 5 defense genes. These results suggest that overexpression of TaRIM1 up-regulates, most likely activates, the expression of a subset of, at least the above-tested 5, defense-related genes. Many papers document that transcription factors firstly bind to specific DNA sequences (*cis*-acting elements) in target genes, and then modulate the transcription levels of these genes^10^,^26^,^27^,^28^,^33^,^34^,^35^,^39^. To address how TaRIM1 up-regulates the expression of the 5 afore-tested defense genes, we analyzed the promoter sequences of the examined 5 defense genes in wheat. The promoter sequences of these 5 defense genes, including *Defensin, PR10, PR17*c, *nsLTP1*, and *Chit1*, contain 2–4 kinds of MBS *cis*-acting elements. Our EMSA results prove that TaRIM1 indeed binds to these MBS *cis*-acting elements (Fig. 4). Thus, we deduce that the TaRIM1 transcription factor interacts with the promoters of these defense genes and then activates the expression of these genes. Consequently, the expression change of a range of wheat defense-related genes regulated by TaRIM1 probably results in enhanced resistance in transgenic wheat to sharp eyespot caused by *R. cerealis* infection.

In conclusion, TaRIM1, a wheat MYB gene induced by *R. cerealis* infection, was identified and its functional role was dissected. TaRIM1 encodes an activator-type R2R3-MYB transcription factor TaRIM1. TaRIM1 positively contributes to wheat resistance response to *R. cerealis* infection through regulation of the expression of a range of defense-related genes. *TaRIM1* is a candidate gene for breeding wheat varieties with resistance to sharp eyespot caused by *R. cerealis* infection. This study provides novel insight into characteristics and functional roles of the MYB members in plants’ defense responses.

## Methods

### Plant and fungal materials, and treatments

*R. cerealis*-resistant wheat line CI12633 was used in cloning and VIGS analysis. The spring wheat cultivar Yangmai16 displaying susceptible was used as transformation recipient. Three resistant lines of RILs (cross of resistant wheat cultivar Shanhongmai and highly-susceptible wheat cultivar Wenmai 6), provided by Prof. Jizeng Jia in our Institute, were used in RNA-Sequencing.

The fungus *R. cerealis* Jiangsu-prevailing strain R0301 and North-China high-virulence strain WK207 were provided by Profs Huaigu Chen and Shibin Cai (Jiangsu Academy of Agricultural Sciences, China,) and Prof. Jinfen Yu (Shandong Agricultural University), respectively.

Wheat plants were grown in a 15 h light (~22 °C)/9 h dark (~10 °C) regime. The treatments were conducted according to the protocol by Zhu *et al*.^31^.

### RNA extraction and cDNA synthesis

Total RNA was extracted using TRIZOL reagent (Qiagen, China) and subjected to DNase I (Takara, Japan) digestion and purification. The first-strand cDNA was synthesized using 2-μg purified RNA, AMV reverse transcriptase and AP primer (5′-GGCCACGCGTCGACTAGTACTTTTTTTTTTTTTTTTT-3′) and Oligo dT primer according to the manual (Takara, Japan).

### Cloning of cDNA and genomic DNA full-length sequences of *TaRIM1*

After inoculation with *R. cerealis* WK207 for 4 or 10 d, RNAs extracted from the infected base stems and sheaths of three resistant lines of the RILs were deeply sequenced. The further transcriptiomic analyses were performed by Bioinformatics to identify up-regulated genes (Data unpublished). Among them, one with no. Traes_6BL_E5A9546C9 is homologous to the *TaMYB33* sequence, thus this corresponding gene was named *TaRIM1*.

To obtain the *R. cerealis*-resistance-related sequence of *TaRIM1*, used as the template to the full-length cDNA of *TaRIM1* was obtained from infected stems’ cDNA of resistant wheat CI12633 by two rounds of 3′RACE reactions. In 1^st^ round, the primers TaRIM1F-F1 (5′-GCAGCATTTACCTTCGGAC-3′) and AUAP (5′-GGCC ACGCGTCGACTAGTAC-3′) were used. The primers TaRIM1F-F2 (5′-CGTCAACA CACTGAGCAATC-3′) and AUAP (5′-GGCCACGCGTCGACTAGTAC-3′) were used in 2^nd^ round. Genomic sequence of *TaRIM1* was obtained from CI12633 genomic DNA by 2 round PCR amplifications and 2 pairs of primers (TaRIM1F-F1: 5′-GCAG CATTTACCTTCGGAC-3′, TaRIM1F-R1: 5′-AACGATTATTGTTCCCTTCACA-3′, TaRIM1F-F2: 5′-CGTCAACACACTGAGCAATC-3′, and TaRIM1F-R2: 5′-AAACTA GCCGAGGAGCCG-3′). The purified PCR products were cloned into the pMD-18T vector (Takara) and sequenced.

Sequence blast was performed online using the website (http://blast.ncbi.nlm.nih.gov/Blast.cgi). Protein sequence analysis was performed using SMART (http://smart.embl-heidelberg.de/). The NLS region was predicted by cNLS Mapper (http://nls-mapper.iab.keio.ac.jp/cgi-bin/NLS_Mapper_form.cgi). Phylogenetic tree analysis was performed using MEGA 5.0.

### Subcellular localization of *TaRIM1* in onion epidermal cells

The *TaRIM1* coding region lacking the stop codon was amplified using primers TaRIM1-GFP-Hind III-F (5′-AGTAAGCTTATGGGGAGGTCTCCTTGC-3′, underline represents the *Hin*d III restriction site) and TaRIM1-GFP-BamHI-R (5′-GCGGGATCCAATCTGAGACAAACTCTG-3′, underline represents the *Bam*H I restriction site). The PCR products was digested with restriction enzymes *Hin*d III and *Bam*H I, then sub-cloned in-frame into 5′-terminus of GFP coding region in the p35S:GFP vector, resulting in the TaRIM1-GFP fusion construct p35S:TaRIM1-GFP.

The p35S:TaRIM1-GFP or p35S:GFP alone construct was separately introduced into wheat leaf protoplasts via the PEG-mediated transfection method following the protocol of Yoo *et al*.^40^ or transformed into onion epidermal cell by particle bombardment following Zhang *et al*.^41^. After incubation at 25 °C for 12 h, GFP signals were then observed and photographed using Confocal Laser Scanning Microscopy (Zeiss LSM 700, Germany).

### VIGS-based functional analysis of *TaRIM1*

The specific fragment (no. 576–836 nt) of *TaRIM1* was amplified from CI12633 cDNA by primers TaRIM1-NheIF (5′-ACAGCTAGCTCGTCCGCCACCGATTACT-3′, underline represents *Nhe* I restriction site) and TaRIM1-NheIR (5′-GGCGCTAGCCTC TGCCTAAATCTGAGACAAAC-3′ underline represents *Nhe* I restriction site). PCR products were digested with *Nhe* I, then ligated into the BSMV-γ vector, resulting in recombinant vector BSMV-γ-TaRIM1.

BSMV:TaRIM1 and control virus BSMV:GFP viruses were used to inoculate the CI12633 using protocol following Zhu *et al*.^31^. At 14 d after infection, the fourth leaves of the inoculated seedlings were collected to monitor BSMV infection, the transcription of the BSMV coat protein (*CP*) gene with the specific primers (BSMV-CPF: 5′-TGACTGCTAAGGGTGGAGGA-3′, BSMV-CPR: 5′-CGGTTGAACATCACGAAG AGT-3′) and RT-PCR was used to evaluate if BSMV inoculates wheat plants. At 20 days after BSMV infection, BSMV infected plants were inoculated with the small toothpick fragments harboring the well-developed mycelia of *R. cerealis* WK 207 34. At 45 dpi with *R. cerealis*, ITs were scored, and sharp eyespot symptoms were photographed.

### EMSA on TaRIM1 binding activity to MBS *Cis*-elements

The coding sequence of *TaRIM1* with *Eco*R I and *Xho* I restriction sites was amplified with the primers (GF: 5′-GATGAATTCAAGAGGGGGCCGTGGACG-3′, underline represents *Eco*R I restriction site. GR: 5′-CTACTCGAGCTGGCCGGACGTCTTGGA -3′ underline represents *Xho* I restriction site) and then sub-cloned in-frame into 3′-terminus of GST in pGEX-4T-1 vector. The resulting GST-TaRIM1 recombinant protein was expressed in *Escherichia coli* BL21 cells after induction with 0.3 mM isopropyl-β-D-thiogalactopyranose, and purified using MicroSpin module (GE Amersham).

The primers of 6 *cis*-element probes including 5 MBS and GCC-box were synthesized as the sequences in Table S1 26.

Following a modified EMSA protocol^42^, each probe was mixed with ~2 μg of recombinant GST-TaRIM1 or GST in a binding buffer. Each reaction mixture was incubated on ice for 6 h and loaded onto 8% polyacrylamide gel. After electrophoresis was performed at 100 V for 30 min, the gels were stained with ethidium bromide for visualization of the DNA bands.

### Generation and PCR detection of *TaRIM1*- overexpressing transgenic wheat

ORF of *TaRIM1* was amplified with the primers (TVF: 5′-CAAACTAGTATGGGGAGG TCTCCTTGC-3′, underline represents *Spe* I restriction site, TVR: 5′-CGTGAGCTCCT AAATCTGAGACAAACT-3′, underline represents *Sac* I restriction site) to sub-clone in-frame with a *c-myc* epitope tag in a modified pAHC25-myc vector^31^,^32^, resulting in *TaRIM1*-overexpression transformation vector pUbi:myc-TaRIM1 (Fig. 6a). In predicted transformed plants, the expression of the fused protein gene *myc-TaRIM1* of a *c-myc* epitope tag and *TaRIM1* was driven by a maize ubiquitin (*Ubi*) promoter and terminated by the terminator of the *Agrobacterium tumefaciens* nopaline synthase gene (*Tnos*).

The resulting transformation vector pUbi:myc-TaRIM1 plasmid was transformed into 1,200 immature embryos of wheat Yangmai 16 by biolistic bombardment following Chen *et al*.^34^.

The overexpressing transgene was detected by a PCR product (374 bp) specific to the transgene using specific primers (TaRIM1-TF1: 5′-CGGACAACGAGATCAA GAAC-3′, TaRIM1-TF2: 5′-AGCTTTACATCGGAGGAGTTTCAG-3′ locating in the *TaRIM1* coding region, TaRIM1-TR: 5′-AAAACCCATCTCATAAATAACG-3′ locating in *Tnos*) with 2 round of nested amplifications.

### Western blot analysis of myc-TaRIM1 protein in transgenic wheat

The protein expression of introduced c-myc-TaRIM1 was evaluated by Western blotting. Total proteins were extracted from 0.4 g of ground base stem powders. About 10 μg of total soluble proteins were separated on 12% sodium dodecyl sulfate polyacrylamide gels and transferred to PVDF membrane. The Western blots were incubated with a 2000-fold dilution of c-myc antibody (TransGen Biotech, China) at 4 °C for 12 h, and then with 1000-fold dilution of secondary antibody conjugated to horseradish peroxidase (TransGen Biotech, China) at ~25 °C for 1 h. The expressing myc-TaRIM1 proteins were visualized using the Pro-light HRP Chemiluminescent Kit (TIANGEN Biotech, China).

### qRT-PCR analyses of *TaRIM1* and defense genes

qRT-PCR technique was used to examine the relative transcriptional levels of *TaRIM1* (*TaRIM1*-QF: 5′- CGACTCGTCCTCGTCCAAG-3′, *TaRIM1*-QR: 5′-CGACGACG GCATCGAGTAAT-3′) and five defense-marker genes in *TaRIM1*-silencing and overexpression wheat plants as well as their control wheat plants. These defense genes include *Defensin* (Defensin-Q-F: 5′-ATGTCCGTGCCTTTTGCTA-3′, Defensin-Q-R: 5′-CCAAACTACCGAGTCCCCG-3′), *PR10* (PR10-Q-F: 5′-CGTGGAGGTAAACGA TGAG -3′, PR10-Q-R: 5′-GCTAAGTGTCCGGGGTAAT-3′), *PR17c* (PR17c-Q-F: 5′-ACGACATCACGGCGAGGT-3′, PR17c-Q-R: 5′-CACGGGGAAAGAGAGGATGA-3′), *nsLTP1* (nsLTP1-Q-F: 5′-ATGCGGGTTGGCGTGAAG-3′, nsLTP1-Q-R: 5′-TGT TGCGGTGGTAGGTTGTTG-3′) and *chitinase1* (Chit1-Q-F: 5′-ATGCTCTGGGACC GATACTT-3′, Chit1-Q-R: 5′-AGCCTCACTTTGTTCTCGTTTG-3′). qRT-PCR was performed (95 °C for 5 min, 41 cycles of 95 °C for 15 s and 60 °C for 31 s) using SYBR Green I Master Mix (Takara) using ABI PRISM 7300 detective system according to the manufacturer’s instruction. The relative transcript levels were calculated using the 2^−ΔΔCT^ method^43^, where the wheat *actin* gene (NCBI accession no. BE425627) with the primers ActA: (5′-CACTGGAATGGTCAAGGCTG-3′) and ActB (5′-CTCCATGTC ATCCCAGTTG-3′) was used as the internal reference.

### Assessment on wheat response to *R. cerealis*

At least 10 plants for each wheat line were inoculated with developed mycelia of *R. cerealis* following the protocol of Wei *et al*.^44^. In T_1_ generation, *R. cerealis* WK207 was used to inoculate plants, whereas *R. cerealis* isolate R0301 was used to inoculate T_2_ and WT plants. ITs and disease index for wheat line were evaluated at ~47 dpi according to Wei *et al*.^44^.

## Additional Information

**How to cite this article**: Shan, T. *et al*. The wheat R2R3-MYB transcription factor TaRIM1 participates in resistance response against the pathogen *Rhizoctonia cerealis* infection through regulating defense genes. *Sci. Rep*. **6**, 28777; doi: 10.1038/srep28777 (2016).

## Supplementary Material

Supplementary Information

## Figures and Tables

**Table 1:** Sharp eyespot responses of *TaRIM1*-overexpression and wild-type wheat lines^a^.

Lines	IT in T_1_	DI in T_1_	IT in T_2_	DI in T_2_
MO1	1.43**	28.60**	1.00**	20.00**
MO2	1.00**	20.00**	1.00**	20.00**
MO3	1.00**	20.00**	1.00**	20.00**
MO4	1.80**	36.00**	1.67**	33.40**
MO5	1.58**	31.60**	1.00**	20.00**
WT	2.75	55.00	2.42	48.40

^a^MO1 to MO5 represent 5 *TaRIM1*-overexpression transgenic wheat lines, respectively. IT represents infection type. DI indicates disease index of sharp eyespot. T_1_ indicates T_1_ generation. T_2_ indicates T_2_ generation. Response value each line is the mean of at least 10 plants in each generation. Statistically significant differences between each line of *TaRIM1*-overexpression wheat (MO1 to MO5) and WT (non-transformation) recipient wheat Yangmai 16 were determined using Student’s *t*-test (**P < 0.01).

**Table 2:** MBS sequences in promoters of defense genes regulated by *TaRIM1*.

Gene Name	Accession number	Site Name	Position	Sequence
*Defensin*	CA630387	MBS1	−765 to −760	CTGTTA
		RT1	−715 to −711	AACGG
		St1R	−591 to −586	GGATAA
		AC1	−373 to −368(−)	CCAACC
*PR10*	CA613496	St1R	−1777 to −1773	GGATA
		MBS1	−1608 to −1602(−)	CGGTTG
		RT1	−141 to −137(−)	AACGG
		AC1	−811 to −804	CACCTAAC
		AC1	−790 to −785	CCAACC
*PR17c*	TA65181	St1R	−58 to −54	GGATA
*nsLTP1*	TC411506	MBS1	−86 to −81(−)	CTGTTA
*Chit1*	CA665185	RT1	−893 to −888	AACGG
		AC1	−772 to −767(−)	CCTACC
		MBS1	−741 to −736	CGGTTG
		MBS1	–249 to –244(−)	CTGTTA
