# Supplementary material for: The wheat R2R3-MYB transcription factor TaRIM1 participates in resistance response against the pathogen Rhizoctonia cerealis infection through regulating defense genes
Source: Sci Rep. 2016 Jul 1;6:28777. doi: 10.1038/srep28777 (PMC4929490; doi:10.1038/srep28777)
Supplement: Supplementary Information [file srep28777-s1.doc]

**Title: The wheat R2R3-MYB transcription factor TaRIM1 participates in resistance response against the pathogen *Rhizoctonia cerealis* infection through regulating defense genes**

**Authors: Tianlei Shan, Wei Rong, Huijun Xu, Lipu Du, Xin Liu, Zengyan Zhang***

**Institution:** The National Key Facility for Crop Gene Resources and Genetic Improvement, Institute of Crop Science, Chinese Academy of Agricultural Sciences, Beijing 100081, China

***Corresponding author:** Zengyan Zhang; Telephone: +86-10-82108781;

Fax: +86-10-82105819; E-mail: [zhangzengyan@caas.cn](mailto:zhangzengyan@caas.cn)

**Table S1 The primers’ sequences of 6 tested probes with *cis-*elements in EMSA**

| *cis*- element | primers |
| --- | --- |
| ACⅠ | 5’-AGTCGACCCACCTACCGCGGATCCTCGACCCACCTACCGTCGATC-3’  5’-GATCGACGGTAGGTGGGTCGAGGATCCGCGGTAGGTGGGTCGACT-3’ |
| MBS1-Bz | 5’-TGCCAGTCGCAGTTAGACGTGCCAGTCGCAGTTAGACGTGC-3’  5’-CACGTCTAACTGCGACTGGCACGTCTAACTGCGACTGGCA-3’ |
| MBS1-W | 5’-AGTCWRXCTGTTGCTGCAWRXCTGTTGCTGCATG-3’  5’-CATGCAGCAACAGXRWTGCAGCAACAGXRWGACT-3’ |
| RT1 | 5’-ATACCAGTCCCCAACGGCTAGCCAGTCCCCAACGGCTAGTTTAACC-3’  5’-GGTTAAACTAGCCGTTGGGGACTGGCTAGCCGTTGGGGACTGGTAT-3’ |
| St1R | 5’-AGTCGACGTGGATAATGGATCCTCGAGTGGATAATTCGATC-3’  5’-GATCGAATTATCCACTCGAGGATCCATTATCCACGTCGACT-3’ |
| GCC-box | 5’-AATTCATAAGAGCCGCCACTCATAAGAGCCGCCACTCCC-3’  5’-GGGAGTGGCGGCTCTTATGAGTGGCGGCTCTTATGAATT-3’ |
